# Drug repurposing candidates to treat core symptoms in autism spectrum disorder

**DOI:** 10.3389/fphar.2022.995439

**Published:** 2022-09-12

**Authors:** Elise Koch, Ditte Demontis

**Affiliations:** ^1^ Norwegian Centre for Mental Disorders Research (NORMENT), University of Oslo and Oslo University Hospital, Oslo, Norway; ^2^ The Lundbeck Foundation Initiative for Integrative Psychiatric Research, iPSYCH, Aarhus, Denmark; ^3^ Department of Biomedicine (Human Genetics) and Centre for Integrative Sequencing, Aarhus University, Aarhus, Denmark; ^4^ Center for Genomics and Personalized Medicine, Aarhus, Denmark

**Keywords:** autism spectrum disorder, drug repurposing, protein-protein interactome, network medicine, genetics

## Abstract

Autism spectrum disorder (ASD) is characterized by high heritability and clinical heterogeneity. The main core symptoms are social communication deficits. There are no medications approved for the treatment of these symptoms, and medications used to treat non-specific symptoms have serious side effects. To identify potential drugs for repurposing to effectively treat ASD core symptoms, we studied ASD risk genes within networks of protein-protein interactions of gene products. We first defined an ASD network from network-based analyses, and identified approved drugs known to interact with proteins within this network. Thereafter, we evaluated if these drugs can change ASD-associated gene expression perturbations in genes in the ASD network. This was done by analyses of drug-induced versus ASD-associated gene expression, where opposite gene expression perturbations in drug versus ASD indicate that the drug could counteract ASD-associated perturbations. Four drugs showing significant (*p* < 0.05) opposite gene expression perturbations in drug versus ASD were identified: Loperamide, bromocriptine, drospirenone, and progesterone. These drugs act on ASD-related biological systems, indicating that these drugs could effectively treat ASD core symptoms. Based on our bioinformatics analyses of ASD genetics, we shortlist potential drug repurposing candidates that warrant clinical translation to treat core symptoms in ASD.

## Introduction

Autism spectrum disorder (ASD) is a heterogeneous group of neurodevelopmental phenotypes characterized by social and communication deficits along with restrictive behaviors (Lord et al., 2018), most often accompanied with psychiatric comorbidities such as sleep problems, anxiety, depression, ADHD, or aggression and irritability (Lamy and Erickson, 2018; Lord et al., 2018). ASD has a high twin heritability (estimates range from 64–93 % (Tick et al., 2016)) and polygenicity, where both common and rare variants contribute to its etiology (Grove et al., 2019; Satterstrom et al., 2020). The latest genome-wide association study (GWAS) on ASD (Grove et al., 2019) demonstrated differences in the polygenic architecture across clinical subgroups of ASD (childhood autism, atypical autism, Asperger’s syndrome, and other/unspecified pervasive developmental disorders).

Whereas there are no medications currently approved for the treatment of social communication deficits, the main core symptom in ASD (Baranova et al., 2021), most adults and about half of children and adolescents with ASD are treated with psychotropic medications to reduce non-core symptoms such as irritability, hyperactivity, and self-injurious behavior (Madden et al., 2017; Stepanova et al., 2017; Lamy and Erickson, 2018; Lamy et al., 2020). Most commonly used medications in ASD are antipsychotics (used by up to 57 % of children (Jobski et al., 2017) and >65 % of adults (Vohra et al., 2016), ADHD medications (used by up to 45 % of children and 15 % of adults (Jobski et al., 2017)), and antidepressants (used by up to 32 % of children and 43 % of adults (Jobski et al., 2017)). Other medications used to treat non-core symptoms in ASD include alpha-2 agonists and anticonvulsants (Madden et al., 2017; Stepanova et al., 2017). Currently, there are only two medications approved by the United States Food and Drug Administration (FDA) for targeting ASD-associated irritability, the antipsychotics risperidone and aripiprazole (Lamy and Erickson, 2018). In Europe, there are no medications currently approved for treatment of ASD-associated symptoms, though guidelines support the use of risperidone and aripiprazole (Howes et al., 2018; Lamy et al., 2020). However, these medications have considerable limitations such as serious side effects including antipsychotic-induced weight gain (Barton et al., 2020) and extrapyramidal symptoms (Cohen et al., 2012; Barton et al., 2020), or lack of efficacy in ASD (Bowker et al., 2011). Individuals with ASD are more vulnerable to side effects of psychopharmacological agents than age-matched individuals without ASD (Accordino et al., 2016), but research on pharmacological management of ASD-associated symptoms is limited to studies with small sample sizes and heterogeneous ASD subgroups (Lamy and Erickson, 2018).

The clinical and genetic heterogeneity of ASD complicates the development of pharmacologic treatments (Bowers, Lin and Erickson, 2015), which necessitates the use of new approaches to identify novel treatment options for ASD. Drugs which target e.g. a receptor or an enzyme encoded by a gene in which genetic variants associate with the target disease have a higher success rate in the drug development pipeline (Nelson et al., 2015). To identify such drugs, interactions of protein products from disease risk genes can be studied within gene networks. The important concept that the network approach embodies is that the effect of a mutation in one gene may not only affect the function of its protein product, but may spread to also impact the function of proteins interacting with it. Therefore, it is important to take protein-protein interactions (PPIs) into account in the effort to reveal genetic disease mechanisms and to identify novel disease genes and drug targets (Menche et al., 2015; Guney et al., 2016). To identify existing drugs that potentially could be used for repurposing to treat conditions other than their original indication, interactions of protein products from disease risk genes can be studied within gene networks (Nabirotchkin et al., 2020). More than 31% of GWAS-associated SNPs are pleiotropic (Watanabe et al., 2019), which provides an explanation why several drugs have been successfully repurposed (Nabirotchkin et al., 2020; Reay and Cairns, 2021). One of the reasons why genes are pleiotropic is that gene products are connected to each other by different mechanisms such as PPIs, thus affecting various biological pathways that can affect several clinical outcomes (Barabási and Oltvai, 2004). Therefore, network-based drug-disease proximity within networks of PPIs can unravel the relationship between drugs and diseases, and serves as a useful tool to identify new indications for approved drugs with known safety profiles (Cheng et al., 2018). However, network proximity is not sufficient for a drug to be effective, as drugs also need to induce the right perturbation in the cell (Gysi et al., 2021). Complex diseases such as ASD are to some extent believed to be caused by variants having a regulatory impact on gene expression (Voineagu, 2012), and drugs that effectively treat their target diseases often revert gene expressions to their normal levels (Pushpakom et al., 2018). To prioritize repurposed drug candidates based on network proximity, gene expression profiles for both the disease and the candidate drugs can be compared to select drug candidates that may counteract disease-associated gene expression perturbations (by down-regulating genes up-regulated in the disease or vice versa).

Improved knowledge of ASD genetics in the context of gene networks incorporating gene expression provides a platform to find existing drugs for repurposing to treat the core symptoms in ASD (Rask-Andersen et al., 2011). In this study, we used a network pharmacology approach and gene expression perturbations to identify potential drugs for repurposing to treat core symptoms in ASD.

## Methods

### Autism spectrum disorder genes

Two different sources were used to define ASD genes; the latest large-scale GWAS on ASD including 18,381 individuals with ASD and 27,969 controls (Grove et al., 2019), and the largest exome sequencing study of ASD (N = 35,584 total samples, 11,986 individuals with ASD) (Satterstrom et al., 2020). From the ASD GWAS (Grove et al., 2019), all genes listed in (Grove et al., 2019) (29 genes near GWAS-significant hits) and STable 10 (Grove et al., 2019) (25 genes from MAGMA gene-based association) were included, resulting in 54 GWAS-identified ASD genes. Of 102 ASD risk genes identified in the whole exome study (Satterstrom et al., 2020), 7 genes were already included from the GWAS (Grove et al., 2019), resulting in 149 ASD candidate risk genes in total (Supplementary Table S1).

### The human protein interactome

For network analyses, the human interactome (Fang et al., 2021) was used. This interactome was constructed from data of 15 commonly used databases, focusing on high-quality protein-protein interactions (PPIs) as follows: Physical PPIs tested by high-throughput yeast-two-hybrid (Y2H) screening system; literature-curated PPIs followed by affinity-purification mass spectrometry (AP-MS), Y2H, and literature-derived low-throughput experiments; physical PPIs derived from protein three-dimensional structures; kinase-substrate interactions by literature-derived low-throughput and high-throughput experiments; and signaling networks by literature-derived low-throughput experiments (for details see Fang et al., 2021). This interactome consists of 17,706 unique proteins (nodes) interconnected by 351,444 PPIs (edges or links), resulting in 346,330 PPIs after removing self-loops. Network figures were created using Cytoscape (Shannon et al., 2003), where nodes refer to genes or drugs, and edges refer to gene-drug interactions or gene-gene interactions through identified PPIs between gene products (proteins).

### Autism spectrum disorder network

As most approved drugs do not target disease proteins, but bind to proteins in their network vicinity (Yildirim et al., 2007), we defined an ASD network including not only the ASD genes that were defined as described above, but also genes in their immediate network proximity. To define the ASD network, we used the method network propagation (Köhler et al., 2008; Vanunu et al., 2010; Carlin et al., 2017), implemented in the Cytoscape application Diffusion (Carlin et al., 2017). Starting with a chosen set of input proteins, information from their PPIs is transferred to all other proteins in the interactome and received from them through an iterative process. Network proximity between proteins is scored depending on their PPIs, where higher diffusion output values relate to higher relatedness to the input proteins (Köhler et al., 2008; Vanunu et al., 2010; Carlin et al., 2017). Genes defined as ASD genes were used as input query genes, and the top 1% of proteins from the diffusion output were included in the ASD network. To examine enrichment in gene ontology annotations for genes in the ASD network, ToppGene (Chen et al., 2009) (last updated 2021–03–29) was used. We included the gene ontology annotation categories molecular function, biological process, cellular component, and pathways, as well as disease. Within each category, a Bonferroni-corrected *p*-value threshold of 0.05 was used.

### Drug target network

The drug-gene interaction database (DGIdb) (Freshour et al., 2021) (version 4.2.0, last updated 2020–10–21) was used to identify drug-gene interactions between approved drugs and genes in the ASD network. The DGIdb provides information on drug-gene interactions from 22 sources that are aggregated and normalized (for description of sources in DGIdb, see Freshour et al., 2021).

### Gene expression perturbation profiles

For the drugs interacting with genes in the ASD network, we utilized gene expression data (drug versus no drug) to evaluate if these drugs modulate the activity of the genes in our network. To determine each drug’s gene expression perturbation profile, we retrieved gene expression data from the Connectivity Map (CMAP) database (Lamb et al., 2006; Subramanian et al., 2017), extracted from the Phase 2 data release of the Library of Integrated Cellular Signatures (LINCS) in GEO series GSE70138 (GSE70138_Broad_LINCS_Level5_COMPZ_n118050x12,328_2017–03–06.gctx.gz available at https://www.ncbi.nlm.nih.gov/geo/query/acc.cgi?acc=GSE70138) using the R-package cmapR (Enache et al., 2019) in R version 4.0.3.

To evaluate ASD-associated gene expression perturbations, genetically regulated gene expression was imputed in 13 brain tissues from GTEx (version 8) (Barbeira et al., 2021) using MetaXcan (Barbeira et al., 2018) implemented in the R-package metaxcanr (https://github.com/drveera/metaxcanr) in R version 4.0.3. MetaXcan imputes the genetically regulated gene expression using summary statistics from GWAS. MetaXcan first predicts gene expression levels based on reference transcriptome data (from GTEx) and then estimates the correlation between the gene expression levels and a phenotype (ASD) using GWAS summary statistics. For MetaXcan, we used the latest ASD GWAS (Grove et al., 2019) as input and gene expression was imputed using high-performance gene expression prediction models trained using elastic net regression (downloaded from http://predictdb.org) on 13 brain expression data sets from GTEx and covariance matrices calculated from 503 individuals with European ancestry from the 1000 Genomes project (The 1000 Genomes Project Consortium, 2015).

To evaluate if the drug repurposing candidates could change ASD-associated gene expression perturbations (whether they down-regulate genes up-regulated in ASD or vice versa), the Spearman correlation ρ between the drug-induced perturbations and the ASD-associated perturbations in genes within the ASD network was calculated for each drug, where negative correlation coefficients indicate that the drug could reverse ASD-associated gene expression changes.

## Results

Of the 149 ASD genes from the ASD GWAS (Grove et al., 2019) and the ASD exome sequencing study (Satterstrom et al., 2020), 147 were present in the human protein interactome (Fang et al., 2021) (Supplementary Table S1).

### Autism spectrum disorder network

**FIGURE 1:**
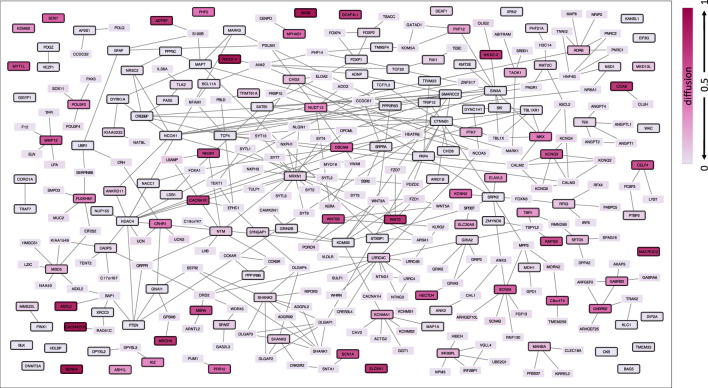
ASD network defined by network propagation. The diffusion output values from the input genes are indicated by different brightness, where darker colors refer to higher diffusion values and thus higher relatedness via protein interaction within the human protein interactome. ASD genes are highlighted with a black node border paint.

To identify genes in the immediate network proximity of ASD genes, we performed a network propagation analysis with all ASD genes (N = 147) as input query, and chose the top 1% of genes from the diffusion output. In total, 323 genes were included in the ASD network (147 ASD genes and 176 ASD-related genes), shown in Figure 1. The genes included in the ASD network and the corresponding diffusion output values as well as their node degrees can be found in Supplementary Table S2. Genes in the ASD network were enriched for biological processes involved in synaptic signaling and brain development and pathways related to the neuronal system, even when excluding ASD genes (Supplementary Table S3). Using gene expression data from 13 brain tissues from GTEx, ASD-associated gene expression could be imputed for 207 out of the 323 genes in the ASD network. The ASD-associated gene expression values (z-scores) can be found in Supplementary Table S4.

### Drug repurposing candidates

**FIGURE 2:**
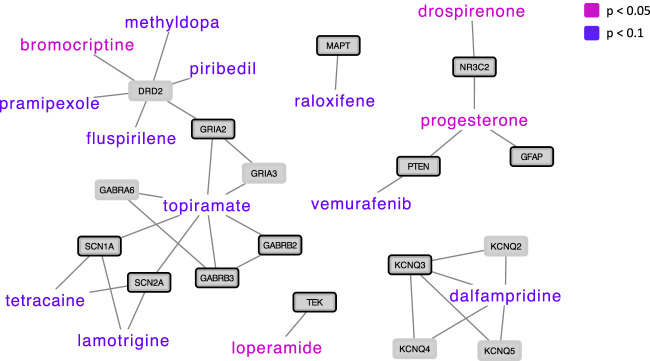
Drug repurposing candidates for ASD based on drug-induced versus ASD-associated gene expression, shown with their protein interaction partners in the ASD network. Drugs whose gene expression perturbation profile was negatively correlated with ASD-associated gene expression perturbations at a *p*-value < 0.05 are presented in pink (*N* = 4), and drugs whose expression perturbation profile was negatively correlated with ASD-associated gene expression perturbations at a *p*-value < 0.1 are presented in violet (*N* = 10). ASD genes are highlighted with a black node border paint.

From the DGIdb (Freshour et al., 2021), drug-gene interactions were identified between 439 approved drugs and 68 genes in the ASD network. Of the 439 approved drugs, 177 drugs were present in CMAP (Lamb et al., 2006; Subramanian et al., 2017), interacting with 60 genes in the ASD network. Out of the 177 drugs, 4 drugs (loperamide, bromocriptine, drospirenone, and progesterone) showed significant (*p* < 0.05) opposite gene expression perturbations in drug (drug-induced expression) versus ASD (ASD-associated expression) in the 60 genes in the ASD network, and 10 drugs showed opposite gene expression in drug versus ASD at *p* < 0.1 (Figure 2). All 177 drugs, their correlation coefficient (drug-induced expression versus ASD-associated expression) and corresponding *p*-value, their interaction partner in the ASD network as well as drug information collected from the DrugBank (https://go.drugbank.com) (Wishart et al., 2018) are listed in Supplementary Table 5.

## Discussion

In the present study, we have identified existing drugs that could potentially be used for repurposing to address core symptoms in ASD. First, we defined an ASD network via network-based methods, and studied the genes in this network in relation to interactions with approved drugs. Then, we selected drug repurposing candidates that could change ASD-associated gene expression perturbations and identified various drugs that may potentially be repurposable to address the core symptoms in ASD.

We identified four drugs showing significant (*p* < 0.05) opposite gene expression perturbations in drug (drug-induced expression) versus ASD (ASD-associated expression) in genes in the ASD network; Loperamide, bromocriptine, drospirenone, and progesterone.

The widely used antidiarrheal medicine loperamide is a μ-opioid receptor agonist that has no central nervous system (CNS) related side effects when used for a short period of time at therapeutic doses (Malinky, Lindsley and Han, 2021). In addition, loperamide has become known as the “poor man’s methadone”, as substance-dependent people have been using loperamide at far higher doses than the recommended dose (2–8 mg/day) as a self-treatment of opioid withdrawal symptoms (Borron et al., 2017; Lasoff et al., 2017). However, the μ-opioid receptors do not only modulate analgesic and rewarding properties of opioids, but they also play a critical role in modulating social behavior in both humans and animals (Julie et al., 2018; Meier et al., 2021). Interestingly, μ-opioid receptor deficient mice show behavioral and social deficits similar to those observed in individuals with ASD, and μ-opioid receptor agonists restore social interaction deficits in rodents (Julie et al., 2018). Thus, the identification of loperamide as a drug repurposing candidate for ASD is consistent with its potential to modulate social behavior in ASD. In addition, it may have favorable gastrointestinal effects in individuals with ASD, as gastrointestinal symptoms are common in ASD (Madra, Ringel and Margolis, 2021).

We further identified the female sex hormone progesterone and the progestin drospirenone, both used as contraceptives (Wishart et al., 2018). Increased testosterone exposure during pregnancy has been associated with ASD development (Knickmeyer and Baron-Cohen, 2002; Auyeung et al., 2015), and testosterone levels have been positively associated with core ASD symptoms such as social anxiety and deficits in social and language developments in individuals with ASD (Crespi, 2016; Ostatníková et al., 2020). Less is known about female sex hormones and their role in ASD symptoms, but disruptions in estrogen signaling have been described in ASD (Crider et al., 2014), and it has also been reported that prenatal estrogen levels were elevated in boys who developed ASD (Alexandros et al., 2020). Interestingly, it has been suggested that low maternal progesterone levels during pregnancy may be related to the development of ASD (Whitaker-Azmitia et al., 2014). In addition, progesterone levels have been positively associated with cognitive performance in healthy individuals (Henderson, 2018).

Bromocriptine, another drug that showed significant opposite gene expression perturbations in drug versus ASD, is a dopamine D2 receptor agonist used for the treatment of prolactin-related conditions (Kvernmo et al., 2006). Two early studies investigating clinical effect of bromocriptine in ASD showed some beneficial effects on global autistic symptoms scales (Dollfus, 1992; Dollfus et al., 1993). In these studies, the effect of bromocriptine on ASD was compared with the dopamine D2 receptor antagonist amisulpride, showing that both drugs had beneficial effects on ASD, with bromocriptine showing predominantly reductions in motor hyperactivity and attention symptoms. The authors speculated that these complementary clinical effects of a dopamine agonist and a dopamine antagonist might be related to similar actions on dopamine autoreceptors, regulating the dopaminergic hyperactivity that has been postulated in ASD (Dollfus, 1992; Dollfus et al., 1993). Although dopaminergic dysfunction in ASD has been widely reported, especially in the midbrain dopaminergic system, the mechanisms are not fully understood (Pavăl and Miclutia, 2021; Mandic-Maravic et al., 2022). Interestingly, both dopamine D2 receptor agonists (pramipexole, piribedil) and an antagonist (the antipsychotic drug fluspirilene) were among the drugs showing opposite gene expression perturbations in drug versus ASD at *p* < 0.1 and interacting with the dopamine D2 receptor gene. Of note, the dopamine D2 receptor gene was not already defined as ASD risk gene, but in our network analyses it was identified as being closely related to ASD risk genes and included in our ASD network. Antipsychotics that are commonly used to reduce non-core ASD symptoms showed either non-significant opposite gene expression perturbations in drug versus ASD (such as aripiprazole) or non-significant positive correlations between drug-induced gene expression perturbations and ASD-associated gene expression (such as risperidone).

Other drugs that showed opposite gene expression perturbations in drug versus ASD at *p* < 0.1 included the anticonvulsants lamotrigine and topiramate, the selective estrogen receptor modulator raloxifene, the anti-cancer drug vemurafenib, the anesthetic agent tetracaine, and dalfampridine (used in Multiple Sclerosis). Anticonvulsants are already used in ASD showing some beneficial effects (Coleman et al., 2019). Dalfampridine has shown procognitive effects in patients with multiple sclerosis (Korsen et al., 2017), and raloxifene in combination with antipsychotics has shown beneficial effects on positive, negative, and cognitive symptoms in both women and men with schizophrenia (Weickert et al., 2015; McGregor et al., 2017; Gogos et al., 2019).

Positive correlations between drug-induced gene expression perturbations and ASD-associated gene expression in the genes in our ASD network indicate that the drug may increase ASD-related gene expression perturbations thereby probably worsen ASD symptoms. Drugs whose gene expression perturbation profile was significantly (*p* < 0.05) positively correlated with ASD-associated gene expression included fluoxetine and epinephrine, and drugs whose gene expression perturbation profile was positively correlated with ASD-associated gene expression at *p* < 0.1 included sumatriptan and metformin. Epinephrine has long been suggested to be involved in the etiology of ASD, and plasma levels of epinephrine and norepinephrine may be elevated in autistic children (Launay et al., 1987). In a recent review summarizing the effectiveness of the selective serotonin reuptake inhibitor (SSRI) fluoxetine in ASD, it was concluded that fluoxetine may be effective in treating repetitive behaviors and irritability, while dose titrating triggers impulsive behavior, hyperactivity, irritability, and sleep disturbance (Launay et al., 1987). While it has been suggested that the anti-diabetic drug metformin may have procognitive effects (Ying et al., 2014), a study in ASD investigating the effects of metformin on memory function did not show any beneficial effects, and while no memory measures differed significantly between participants randomized to metformin versus placebo, the metformin group showed less improvement in verbal learning compared to the placebo group (Aman et al., 2018). Sumatriptan is a 5HT_1B/D_ receptor agonist commonly used to treat migraine attacks (Tfelt-Hansen and Hougaard, 2013). Studies evaluating the role of the 5HT_1D_ receptor in ASD have shown that individuals with ASD have a higher sensitivity of the 5HT_1D_ receptor, which may be related to the severity of repetitive behaviors (Hollander et al., 1999; Novotny et al., 2000). However, sumatriptan’s effects on the CNS are not well-studied, because it has been long assumed that triptans do not penetrate the CNS (Tfelt-Hansen, 2010).

It should be noted that it is not known if the drugs whose gene expression perturbation profile was positively correlated with ASD-associated gene expression worsen ASD symptoms, and neither is it known if the drugs showing opposite gene expression perturbations in drug versus ASD effectively counteract ASD-associated gene expression. It should also be noted that the correlation coefficients shortlisting drugs were quite low, and the corresponding *p*-values are reported at an uncorrected level. As it is not known if the shortlisted drugs effectively treat the core symptoms in ASD, these potential drug repurposing candidates warrant clinical translation to evaluate their effectiveness in ASD. Moreover, the drug-induced gene expression profiles were based on experiments in cancer cell lines. However, such data have been shown to be of value for repurposing drugs even for non-cancer diseases, as shown by topiramate, an anticonvulsant drug that was identified to be potentially repurposable for inflammatory bowel disease (IBD), which has been validated *in vivo* (Dudley et al., 2011). Finally, it should be noted that three of the four drugs showing significant (*p* < 0.05) opposite gene expression in drug versus ASD interact with only one protein in our ASD network. Network pharmacology analyses have demonstrated that drugs acting on a single drug target within a disease network are often not effective (Csermely et al., 2005; Korcsmáros et al., 2007). However, the development of multi-target drugs affecting complex systems remains challenging (Muhammad et al., 2018; Yadav and Tripathi, 2019). Here, we combine network-based analyses with gene expression profiles and shortlist drugs that potentially could be used for repurposing to treat the core symptoms in ASD. These results require follow-up experiments and finally clinical trials to enable clinical translation.

## Conclusion

Based on our bioinformatics analyses of ASD genetics, we shortlist potential drug repurposing candidates that warrant clinical translation to treat ASD-specific symptoms.

## Data Availability

The datasets presented in this study can be found in online repositories. The names of the repository/repositories and accession number(s) can be found in the article/Supplementary Materials.
