# Supplementary figures and images for: Drug repurposing candidates to treat core symptoms in autism spectrum disorder
Source: Front Pharmacol. 2022 Sep 12;13:995439. doi: 10.3389/fphar.2022.995439 (PMC9510394; doi:10.3389/fphar.2022.995439)

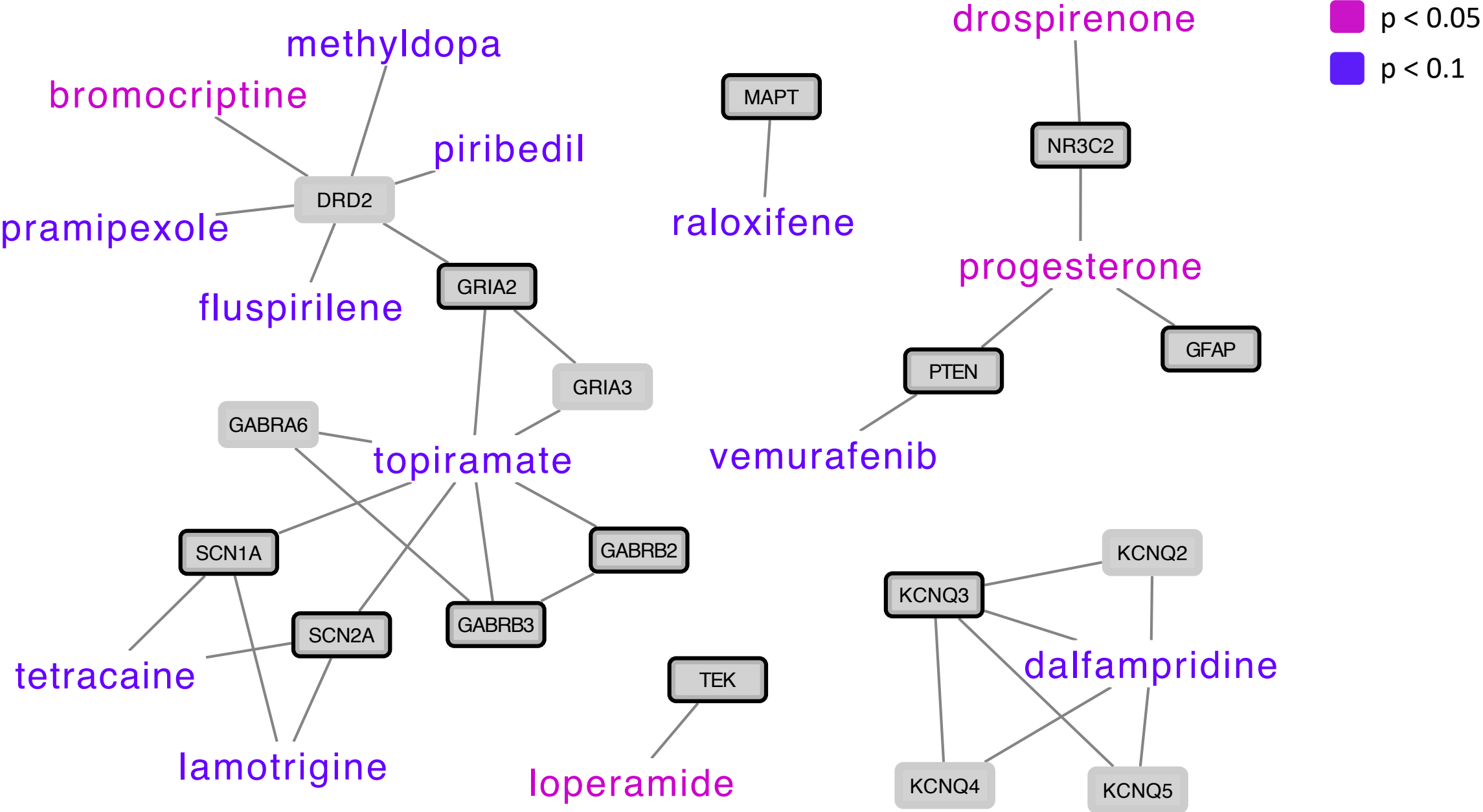

Supplement: Supplementary file 3 [file Image2.PDF]

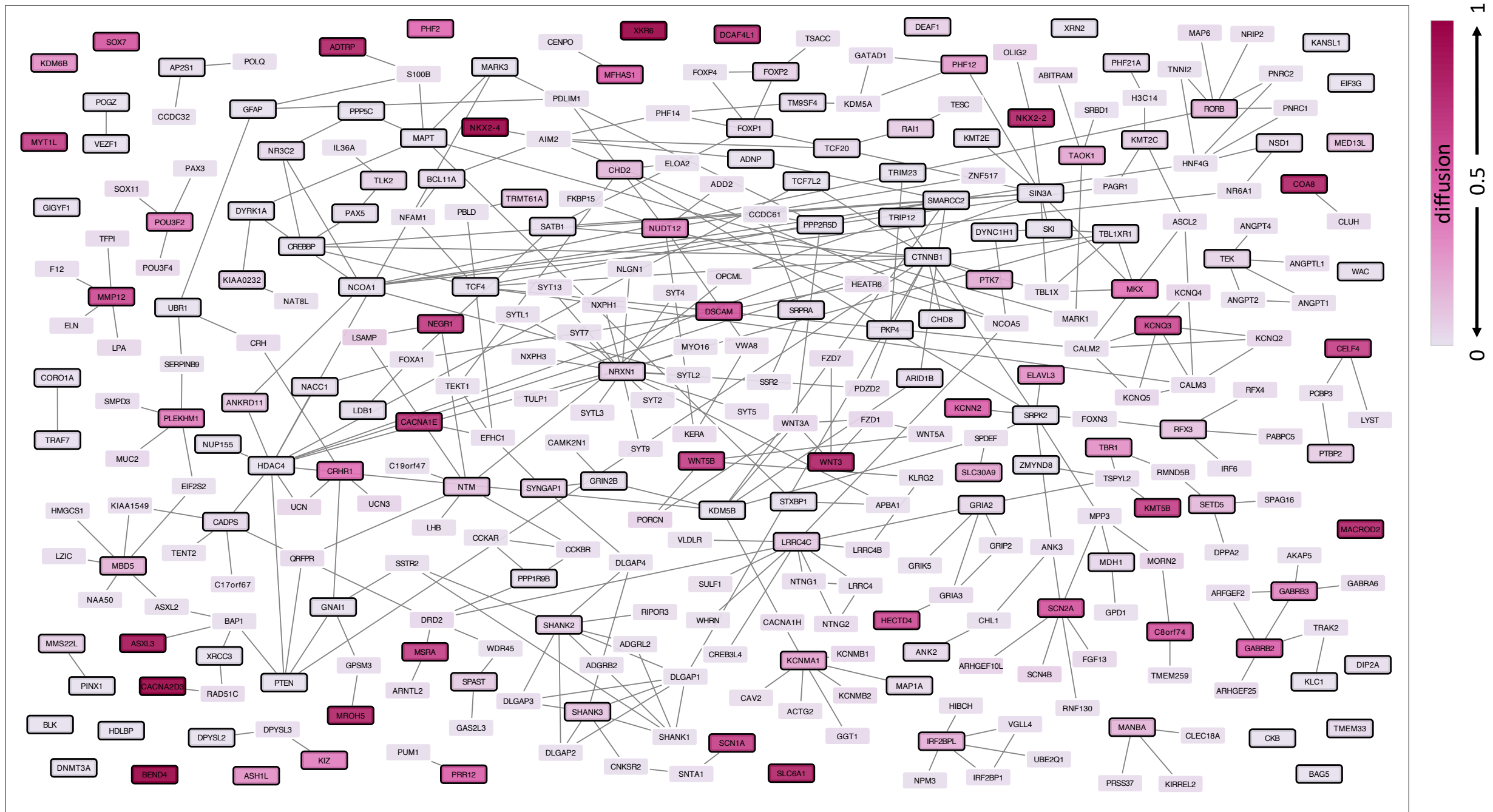

Supplement: Supplementary file 7 [file Image1.PDF]
